# Improving Uptake of Emergency Department-initiated Buprenorphine: Barriers and Solutions

**DOI:** 10.5811/westjem.2022.2.52978

**Published:** 2022-07-11

**Authors:** Timothy D. Kelly, Kathryn F. Hawk, Elizabeth A. Samuels, Reuben J. Strayer, Jason A. Hoppe

**Affiliations:** 1 Indiana University Emergency Medicine Residency, Indianapolis, Indiana; 2 Yale School of Medicine, Department of Emergency Medicine, New Haven, Connecticut; 3 Alpert Medical School of Brown University, Department of Emergency Medicine, Providence, Rhode Island; 4 Maimonides Medical Center, Department of Emergency Medicine, Brooklyn, New York; 5 University of Colorado School of Medicine, Department of Emergency Medicine, Aurora, Colorado

## Abstract

Emergency departments (ED) are increasingly providing buprenorphine to persons with opioid use disorder. Buprenorphine programs in the ED have strong support from public health leaders and emergency medicine specialty societies and have proven to be clinically effective, cost effective, and feasible. Even so, few ED buprenorphine programs currently exist. Given this imbalance between evidence-based practice and current practice, proven behavior change approaches can be used to guide local efforts to expand ED buprenorphine capacity. In this paper, we use the theory of planned behavior to identify and address the 1) clinician factors, 2) institutional factors, and 3) external factors surrounding ED buprenorphine implementation. By doing so, we seek to provide actionable and pragmatic recommendations to increase ED buprenorphine availability across different practice settings.

## INTRODUCTION

Medical treatment for opioid use disorder (OUD) with opioid agonists decreases opioid-specific and all-cause mortality,^1^ human immunodeficiency virus, and hepatitis C transmission,^2^ as well as interactions with the criminal justice system.^3^ Although methadone and buprenorphine, referred to as medication for opioid use disorder (MOUD), are widely recognized to be the most efficacious therapy,^4^ there is a significant treatment gap.^5^ With nearly all (96%) states lacking the capacity to provide MOUD to all appropriate patients,^6^ there is a vital need to improve access for those with OUD who are seeking treatment.

Emergency departments (ED) often serve as the primary access to medical care for underserved populations^7^ and have an essential role in facilitating linkage to the healthcare system and addressing health disparities.^8^ Although addiction treatment has traditionally been perceived to be outside the scope of emergency medicine, emergency physicians (EP) commonly treat patients with OUD, many of whom are often disenfranchised, marginalized, and have less reliable access to healthcare. Expanding the scope of emergency medicine to include initiation of addiction treatment is essential because the ED is where these patients present.^9^,^10^,^11^

Emergency departments are caring for increasing numbers of people with OUD, particularly after an opioid overdose.^10^ Unfortunately, the COVID-19 pandemic has accelerated this phenomenon by interrupting established outpatient behavioral health resources/clinics.^12^,^13^ In the year following an opioid overdose the risk of death exceeds that of other commonly treated ED conditions including chest pain and syncope.^14^,^15^ Recognizing the key role of the ED in the healthcare system, some EDs have developed programs to provide ED buprenorphine and linkage to outpatient MOUD treatment by identifying gaps in treatment access.^16^,^17^,^18^,^19^ Buprenorphine initiated in the ED is associated with increased retention in addiction treatment, decreased rates of self-reported illicit opioid use, and increased cost effectiveness when compared to nonpharmacologic interventions.^16^,^20^ These programs have had considerable success and enjoyed strong support from specialty societies^21^ and national public health leaders.^11^ Even so, adoption of ED buprenorphine programs has been very limited. In 2017, more than 47,000 individuals died of opioid overdose, yet only 5% of EDs offered buprenorphine.^22^,^23^

Barriers and facilitators to implementation of ED buprenorphine treatment have been described previously.^24^,^25^ The ongoing buprenorphine treatment gap demonstrates the need to offer solutions beyond exploring barriers to ED buprenorphine to meaningfully improve access to care. Given that modifying clinical behaviors and organizational practice can be difficult, slow, and resource intensive,^24^,^26^ the gap between evidence-based care and clinical practices necessitates novel and creative approaches to change treatment paradigms. Theoretical models of behavioral change are increasingly being used to effectively guide program implementation and have the potential to more expediently transform clinical practice.^27^,^28^ Here, we use the theory of planned behavior (TPB) to provide guidance in facilitating implementation and adoption of ED buprenorphine initiatives through actionable recommendations to address pivotal individual, organizational, and external barriers.

## CONCEPTUAL FRAMEWORK

Our conceptual framework is based upon TPB, a well-established theory of behavioral change used to describe, predict, and modify human behavior for more than 30 years.^29^ The TPB postulates that behavioral intentions are determined by a person’s attitudes, local norms, and perceived control over a given behavior.^30^ By extension, TPB proposes that the intention to perform any behavior is influenced by 1) personal preference and/or bias, 2) whether or not others in your environment perform the behavior, and 3) the ease (or lack thereof) of performing the behavior. Although there are a multitude of behavioral change theories, TPB is recognized as one of the preeminent frameworks of behavioral change and has a demonstrated track record of effectively promoting the adoption of evidence-based physician decision-making.^31^,^32^,^33^ As a clinical intervention that is evidence-based yet lacking in widespread implementation, ED buprenorphine initiatives would benefit from the application of TBP.

The theory of planned behavior has demonstrated utility in altering the clinical behaviors of emergency physicians. It has been used to understand and contextualize the relative uptake of computed tomography clinical imaging rules^34^ and has also been used to design clinical curriculums that successfully modified the behavioral intentions of EPs.^35^ More recently, Samuels et al and Choo et al used TPB as a framework to inform changes in opioid-related clinical behaviors among clinicians in the ED.^36^,^37^

**Figure 1:**
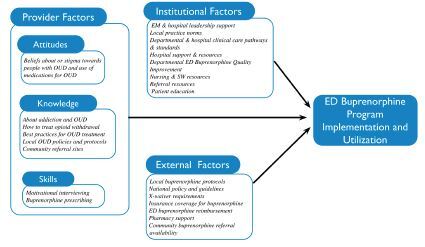
Conceptual diagram. *ED*, emergency department; *EM*, emergency medicine; *OUD*, opioid use disorder.

Although the theory is well known to behavioral psychologists, EPs may be less familiar with it. To provide a more pragmatic and tangible conceptual framework for emergency medicine, we engaged in a deliberative and iterative process to reframe the principles of TPB into 1) clinician factors, 2) institutional factors, and 3) external factors that influence ED buprenorphine program adoption and implementation (Figure 1). This process used three rounds of live discussion, collaboration, and debate, all held over a virtual videoconferencing platform. The proposed theoretical foundation underwent multiple revisions as we sought to maximize its validity and practical applicability. At the end of this process, each author agreed with the chosen theoretical framework. Our conceptual framework mirrors the original conception of TPB and is further informed by our experiences as EPs and administrators of ED buprenorphine programs. We believe that this conceptual approach is appropriately grounded in behavioral change theory but better reflects the unique experiences and demands in EDs.

*Population Health Research Capsule*

What do we already know about this issue?

*Emergency department (ED) buprenorphine is an evidenced-based treatment for opioid use disorder, yet too few EDs offer this important therapy.*

What was the research question?

*Can theoretical models of behavior change help facilitate the increased adoption of ED buprenorphine?*

What was the major finding of the study?

*Theoretical models of behavior change can help more effectively guide efforts to increase ED buprenorphine.*

How does this improve population health?

*Expanding access to ED buprenorphine is essential to providing more effective and equitable care to an underserved and marginalized patient population.*

## CLINICIAN FACTORS

The first step in implementing any new treatment is overcoming clinician-level barriers to adoption, which hinges on individual clinicians concluding that doing this is right for patients. Currently, most EPs do not feel prepared to discuss MOUD with patients.^38^ Educational efforts should highlight the devastating outcomes associated with untreated OUD and nonfatal overdose in ED patients: recent studies have demonstrated between 1 in 8 and 1 in 18 patients who present to the ED with nonfatal overdose die within one year, a mortality rate exceeding that of myocardial infarction and congestive heart failure, which is even more alarming when considering that many of these preventable deaths often occur in young, otherwise healthy people.^14^,^15^

A foundational hurdle in advancing MOUD is overcoming the stigma surrounding addiction and addiction treatment. For generations, addiction has been viewed as a consequence of bad choices resulting from a failure of morals or willpower, instead of being recognized as a disease of altered brain chemistry driven primarily by genetic and environmental factors.^39^,^40^ These socially discrediting attitudes are reinforced by laws and regulations in the United States that criminalize recreational drug use and sequester addiction medicine away from the treatment of every other disease.^41^,^42^ Stigma can be overcome using education, language, and action.

To correct widely repeated untruths such as “buprenorphine replaces one addiction with another” and counter misperceptions about substance use and evidence-based treatment, education is essential. Education can address knowledge gaps regarding the efficacy and effectiveness of medication-based addiction treatment to save lives and return people with OUD to function and health. Replacing stigmatizing language (eg, addict, junkie, user) with person-centered, humanizing language (eg, person with addiction, person with substance use disorder) has a self-perpetuating effect within a department and can contribute to the culture shift often required for success (Table).^43^ These skills and attitudes are effectively taught in classes and workshops, some of which are publicly available; a particularly powerful approach is to ask patients with lived experience to share their stories or use standardized patient actors in an OUD simulation session that allows clinicians to confront their biases and knowledge gaps in a controlled, supportive environment.^44^

Perhaps the most effective way to overcome stigma within a department is to start treating OUD with evidence-based treatment available in EDs, namely buprenorphine. Anecdotally, the immediate feedback loop of watching patients who present in significant distress from withdrawal experience substantial improvement with appropriate treatment can have a meaningful impact on ED staff and operations. Many EPs feel that they lack the knowledge and skills to identify patients who would benefit from ED-initiated buprenorphine and successfully initiate treatment.^24^,^46^ This uncertainty likely arises from a lack of education on buprenorphine’s pharmacology, and the unusual regulations that, until recently, governed its prescription.

Buprenorphine prescription previously required an eight-hour training for physicians and a 24-hour training for nurse practitioners and physician assistants to obtain a Drug Addiction Treatment Act of 2000 (DATA 2000 X) waiver. Recently, the US Department of Health and Human Services (HSS) published new practice guidelines, which exempts clinicians who treat up to 30 patients with buprenorphine from X-waiver training and other requirements pertaining to counseling and ancillary services.^47^ Although enrolling in a waiver training course is an excellent way to gain expertise in using buprenorphine, the legislated training requirement created an unintended barrier.^48^,^49^,^50^ Now, departments can successfully implement effective OUD treatment and ED-initiated buprenorphine by having clinicians register with the Substance Abuse and Mental Health Services Administration to treat 30 or fewer patients at a time.

Clinicians may be reluctant to administer buprenorphine for fear of inducing worsening withdrawal symptoms, an important consideration given buprenorphine’s high-affinity, partial-agonist pharmacology. However, a targeted history and simple assessment can verify the severity of withdrawal using the Clinical Opioid Withdrawal Scale (COWS) to prevent buprenorphine-precipitated withdrawal.^51^ These validated clinical scoring tools can be incorporated into electronic health record (EHR) systems to maximize EPs’ use of and clinical familiarity with buprenorphine, as well as improve communication between clinicians. Patients who have been using methadone are particularly susceptible to buprenorphine-precipitated withdrawal and should not be treated with buprenorphine for 3–5 days after their last dose, and not without a convincingly high COWS score. Emergency department-precipitated withdrawal is a rare event, observed in <1 in a large case series.^52^ Furthermore, in many instances buprenorphine-precipitated withdrawal is effectively treated with higher doses of buprenorphine.^53^ The same approach can be considered in the particularly high-risk group of patients presenting with naloxone-precipitated withdrawal after overdose, who can be given buprenorphine both to relieve withdrawal symptoms and protect them from the toxicity of full agonist opioids, although the evidence for this approach is limited.^54^

Emergency clinicians work in a time-stressed environment and may perceive ED-initiated buprenorphine as a burdensome additional task. However, treatment of opioid withdrawal syndrome with non-agonists (eg, clonidine, promethazine) is less effective and may lead to a protracted ED stay. Patients in severe withdrawal who are treated with buprenorphine will experience rapid relief while simultaneously initiating highly effective treatment for OUD, often without the need to place an intravenous line. Furthermore, by offering tools to effectively manage a patient population that is often perceived as “difficult,” developing proficiency and confidence in treating OUD improves clinician knowledge.^55^

## INSTITUTIONAL FACTORS

On an institutional level, several factors can facilitate the development of an effective program to initiate buprenorphine in the ED. Although there is no single “recipe,” successful programs share several key components; these include the presence of 1) at least one local champion, 2) departmental leadership support, 3) a site-specific protocol, and 4) a clear referral pathway for linkage to outpatient treatment. Many programs also have additional components that support implementation including onsite support staff (social workers and/or patient navigators), screening questions for substance use disorders, clinical decision support pathways which may be integrated with the EHR, and robust quality improvement programs that include feedback on patient linkage to treatment on an individual and group level.^24^,^25^,^38^,^46^

Widespread practice change within a department depends on normalizing institutional best practice expectations and demonstrating that our peers are doing this. A trusted department champion who is willing to provide close to real-time support to clinicians on shift who have questions or concerns can be a powerful tool in closing the local treatment gap. Additionally, leveraging departmental leadership support can be effective. Clear evidence of departmental and institutional support is recognized as among the most important components to building successful programs because leadership philosophy and priorities guide clinical practice, quality improvement priorities, and resource allocation.^24^ Departmental leadership can also be critical to reducing stigma by supporting education on addiction and buprenorphine to clinicians, nurses, techs, social workers, pharmacists, and other ED staff who play an important part in supporting ED OUD treatment. Leadership can facilitate adoption of ED-initiated buprenorphine by providing buprenorphine education specific to the ED setting.^56^,^57^

The overall objective of ED buprenorphine programs is to streamline the delivery of evidence-based care for OUD by minimizing additional clinician effort and workflow disruption through the development of site-specific protocols and referral pathways. Departmental protocols and care pathways are widely used to standardize the delivery of high-quality care for ED patients with sepsis, acute coronary syndromes, and stroke. Protocols effectively set the standard of care for an ED based on the best evidence to date, are agreed upon by local experts and departmental leadership, and typically align with quality measures or best practices as determined by the Centers for Medicare and Medicaid, the American College of Emergency Physicians (ACEP), and other organizations. Furthermore, protocols reduce the cognitive load of clinicians by providing eligibility criteria and direction about patient selection, clinical management, and follow-up. Adapting one of many published ED-specific initiation pathways^58^,^59^ to local operations can help overcome apprehension related to inexperience or unfamiliarity with buprenorphine, decrease treatment variability, and mitigate the stigma for patients who are receiving treatment for substance misuse.

One of the most common concerns about initiating buprenorphine in the ED is the lack of outpatient resources to provide ongoing care.^38^,^46^ Identifying local outpatient treatment resources and establishing reliable referral pathways is a critical role of the local champion, as the optimization often requires an ongoing relationship with key stakeholders at local clinics. These relationships are vital early in the process to provide bidirectional feedback, troubleshoot challenges, and address the needs of patients, EPs and outpatient professionals. Stakeholder input and clinical practice has guided the integration of protocols and clinician decision support in many EDs and has prompted the exploration of automated referrals into the EHR.^60^ A pilot test of a user-centered clinical decision support tool integrated within the EHR more than doubled rates of ED-initiated buprenorphine and naloxone prescribing, and almost doubled the number of physicians who prescribed MOUD.^61^,^62^

## EXTERNAL FACTORS

Federal, state, and local policies outside the hospital and ED also determine whether EPs can provide buprenorphine without difficulty. Key external considerations include prescribing regulations and restrictions, insurance coverage of medications, reimbursement, outpatient treatment availability, and community pharmacy practices. Until recently, buprenorphine was one of the most tightly federally regulated prescription medications in the US.^47^ The recent decision by the HHS to exempt certain clinicians from the full X waiver training is a laudable step toward expanding access to buprenorphine and addiction treatment. Nonetheless, it is important to understand the extent to which previous federal requirements created significant limitations to buprenorphine treatment access, particularly in rural areas.

Approximately 40% of US counties do not have a buprenorphine prescriber, and significant socioeconomic disparities exist in access to buprenorphine and methadone treatment.^6^,^63^,^64^,^65^,^66^ Although a critical step, X waiver exemptions may not translate into increased buprenorphine access if physicians are reticent to prescribe a medication with which they have had minimal previous experience. Given the recent changes in X waiver training requirements, emphasis should be placed on focused training and decision support for clinicians inexperienced in its use. Additionally, some institutions have developed additional “bridge clinics” that address outpatient gaps by stabilizing patients on an initial buprenorphine regimen and facilitating linkage to comprehensive outpatient treatment.^67^ Telehealth addiction treatment is an evolving care solution that could be used to address the OUD treatment gap and has gained traction during the COVID-19 epidemic, but it has not yet been widely available or implemented.^68^,^69^,^70^,^71^,^72^

Patients’ ability to access prescribed buprenorphine is influenced by medication cost and the policies of their local pharmacies. For patients lacking insurance, out-of-pocket costs can be prohibitive.^73^ Depending on insurance coverage, prescribing buprenorphine monotherapy (vs buprenorphine/naloxone) may help decrease these costs. Some insurers require prior authorization for buprenorphine prescriptions, although this practice is prohibited in some states.^74^,^75^ Finally, the availability of buprenorphine at local pharmacies can affect access following ED discharge. Barriers to provision include medication stocking, ability to verify a prescriber’s X waiver, and pharmacist stigma.^76^,^77^,^78^ Developing relationships with local pharmacies may facilitate access and help guide patients to a pharmacy where they can successfully fill prescriptions.

Modifying existing reimbursement structures is another avenue through which EPs, professional societies, and government regulators can influence implementation of ED buprenorphine. There are numerous existing examples of reimbursable clinical actions that support ED buprenorphine. For instance, Screening, Brief Intervention, and Referral to Treatment is frequently used in conjunction with buprenorphine initiation and is reimbursable by Medicare and many state Medicaid programs.^79^ Furthermore, some state Medicaid offices will also reimburse for peer recovery specialists to help engage patients and support seeking treatment and coordinate linkage to outpatient care.^80^

At the national level, there is evidence that payers increasingly value ED buprenorphine initiatives. In the 2020 Centers for Medicare and Medicare Services (CMS) proposed Physician Fee Schedule,^81^ CMS requested comment about whether ED initiation of buprenorphine and referral to treatment should be eligible for separate payment. These encouraging, albeit modest, signs that reimbursement systems will reward ED buprenorphine programs highlights the need for ongoing advocacy from our professional societies. To this end, ACEP has advocated for separate payment of ED buprenorphine initiation, which would incentivize and expand provision of buprenorphine in the ED.^82^ Moving forward, EM professional societies should insist on providing fair and effective reimbursement for such critical clinical interventions.

State and national EM, public health, and treatment organizations have developed strategies to support ED buprenorphine initiation.^83^,^84^ Some cities and states have implemented incentive programs,^85^,^86^ policy guidance,^87^,^88^ or legislative or regulatory requirements.^89^,^90^ Within EM, ACEP partnered with the American Academy of Addiction Psychiatry to produce buprenorphine training tailored to EM,^91^ ED-buprenorphine clinical support applications,^92^ and a quality improvement initiative, the Emergency Quality Network Opioid Initiative.^93^ These resources provide online educational content as well as quality metric reporting to support participating community and academic EDs to improve care for people with OUD.

## DISCUSSION

Using established theory-based models of behavioral change can help identify and address common barriers to building buprenorphine initiation programs in the ED. Informed by our experiences and content expertise, we identified clinician, institutional, and external factors that can promote clinician initiation of ED buprenorphine to treat OUD and opioid withdrawal. Our proposed framework supports recent efforts to use theoretical models of behavioral change to more effectively understand and modify opioid-related ED clinical practices.^36^,^37^

Our approach identifies numerous pragmatic solutions that have the potential to meaningfully increase ED buprenorphine use. Tackling clinician barriers will require personal and professional commitments to the concept that providing buprenorphine initiation is right for our patients. This requires deliberate efforts to reframe language around substance use disorders, improve clinical familiarity with buprenorphine through targeted education and clinical practice interventions, and accept responsibility to decrease the profound stigma associated with treatment. Institutions can support this process by normalizing the process of ED buprenorphine initiation and provide feedback to show that our peers are doing this. Celebrating local champions, promoting effective departmental leadership, establishing departmental guidelines/protocols, and exploring operational interventions to maximize efficiency are just some of the strategies that EDs can employ to this end. Finally, the EM community at large should engage in the legal, sociopolitical, and economic arenas that can facilitate the ability to provide life-saving, evidence-based treatment with buprenorphine. These efforts were instrumental to the recent regulatory shift and offer hope that a permanent legislative fix is feasible with continued engagement and advocacy. From strengthening relationships with local pharmacies to supporting the expansion of substance use disorder treatment in all forms, the EM community should take action to reduce external barriers to allow our clinicians to provide buprenorphine without difficulty.

These recommendations collectively form a comprehensive roadmap, guided by behavioral change theory, to improve ED buprenorphine availability. Although there are many interventions that are likely to help increase ED treatment capacity, the most effective strategy may be one that is multi-faceted, addresses multiple domains, and fits the context of local EDs. These behavioral interventions may be synergistic in nature, and a diversity of interventions may be required to meaningfully increase access to buprenorphine depending upon the practice setting. It is also important to acknowledge that certain EDs may not have the institutional resources to implement some of the more resource-intensive recommendations outlined here. In these instances, it is important to focus on interventions that are obtainable given local resources to build momentum. Given the persistence of the MOUD treatment gap, thoughtful approaches to ED buprenorphine expansion are needed now more than ever.

The most important directions for future study concern which interventions are most effective at promoting ED buprenorphine program adoption, how to effectively implement them at a variety of practice sites, and how to sustain these programs. Most published interventions to date have focused on proving effectiveness and have not attempted to comprehensively address implementation. Finally, this theoretical approach has potential relevance to other EM clinical arenas that are struggling with implementing behavior change.

## LIMITATIONS

This approach has limitations that warrant consideration. Our application of a theoretical model of behavioral change toward ED buprenorphine exclusively uses a single theoretical framework. As evidenced in the process of identifying a specific conceptual framework, there were significant challenges in finding a single model of behavioral change that all authors could relate to. Our work does not intend to suggest that other models of behavior change are less useful in changing attitudes about ED buprenorphine. Additionally, this model is based upon the theory of planned behavior but, albeit intentionally, it is not an exact application of TPB. Our framework does not test the predictive ability of the various TPB constructs, nor does it provide conclusive evidence as to which intervention is most effective. Future research should study the relative effectiveness of various approaches to facilitate ED buprenorphine program implementation.

## CONCLUSION

Initiation of buprenorphine in the ED is an evidence-based treatment for opioid use disorder that must be broadly implemented to address current treatment gaps. A theory of planned behavior approach can identify and offer solutions to common personal, institutional, and external barriers to ED buprenorphine program implementation. Future investigations should examine the effectiveness of interventions specifically guided by behavioral change theory.

## Supplementary Information



## Figures and Tables

**Table:** Replacing stigmatizing language, taken and adapted from the National Institute on Drug Abuse.^45^

Avoid	Employ	Why
Addict User Substance or drug abuser Junkie Alcoholic Drunk Former addict Reformed addict	Person with substance use disorder Person with OUD or person with opioid addiction (when substance in use is opioids) Patient Person with alcohol use disorder Person who misuses alcohol/engages in unhealthy/hazardous alcohol use Person in recovery or long-term recovery Person who previously used drugs	Person-first language. The change shows that a person “has” a problem rather than “is” the problem. The terms avoid negative associations, punitive attitudes, and individual blame.
Habit	Substance use disorder Drug addiction	Inaccurately implies that a person is choosing to use substances or can choose to stop. “Habit” may undermine the seriousness of the disease.
Abuse	For illicit drugs: Use For prescription medications: Misuse Used other than prescribed	The term “abuse” is associated with negative judgments and punishment. Legitimate use of prescription medications is limited to their use as prescribed by the person to whom they are prescribed. Consumption outside these parameters is misuse.
Opioid substitution replacement therapy	Opioid agonist therapy Medication treatment for OUD Pharmacotherapy	It is a misconception that medications merely “substitute” one drug or “one addiction” for another.

*OUD*, opioid use disorder.
