# Supplementary material for: Improving Uptake of Emergency Department-initiated Buprenorphine: Barriers and Solutions
Source: West J Emerg Med. 2022 Jul 11;23(4):461–7. doi: 10.5811/westjem.2022.2.52978 (PMC9391022; doi:10.5811/westjem.2022.2.52978)
Supplement: Supplementary file 1 [file wjem-23-461-s001.docx]

**REFERENCES**

1. Larochelle MR, Bernson D, Land T, et al. Medication for Opioid Use Disorder After Nonfatal Opioid Overdose and Association With Mortality: A Cohort Study. *Ann Intern Med*. 2018;169(3):137-145.
2. Tsui JI, Evans JL, Lum PJ, Hahn JA, Page K. Association of opioid agonist therapy with lower incidence of hepatitis C virus infection in young adult injection drug users. *JAMA Intern Med*. 2014;174(12):1974-1981. doi:10.1001/jamainternmed.2014.5416
3. Russolillo A, Moniruzzaman A, McCandless LC, Patterson M, Somers JM. Associations between methadone maintenance treatment and crime: a 17-year longitudinal cohort study of Canadian provincial offenders. *Addiction*. 2018 Apr;113(4):656-667. doi: 10.1111/add.14059. Epub 2017 Nov 19. PMID: 28987068.
4. Substance Abuse and Mental Health Services Administration (US); Office of the Surgeon General (US). Facing Addiction in America: The Surgeon General’s Spotlight on Opioids [Internet]. Washington (DC): US Department of Health and Human Services; 2018 Sep. Available from: https://www.ncbi.nlm.nih.gov/books/NBK538436/
5. Volkow ND, Frieden TR, Hyde PS, Cha SS. Medication-assisted therapies--tackling the opioid-overdose epidemic. *N Engl J Med*. 2014 May 29;370(22):2063-6. doi: 10.1056/NEJMp1402780. Epub 2014 Apr 23. PMID: 24758595.
6. Jones CM, Campopiano M, Baldwin G, McCance-Katz E. National and State Treatment Need and Capacity for Opioid Agonist Medication-Assisted Treatment. *Am J Public Health*. 2015 Aug;105(8):e55-63. doi: 10.2105/AJPH.2015.302664. Epub 2015 Jun 11. PMID: 26066931; PMCID: PMC4504312.

Zink BJ. Social justice, egalitarianism, and the history of emergency medicine. *Virtual Mentor*. 2010 Jun 1;12(6):492-4. doi: 10.1001/virtualmentor.2010.12.6.mhst1-1006. PMID: 23158453.

1. McKay MP, Vaca FE, Field C, Rhodes K. Public health in the emergency department: overcoming barriers to implementation and dissemination. *Acad Emerg Med*. 2009 Nov;16(11):1132-7. doi: 10.1111/j.1553-2712.2009.00547.x. PMID: 20053233; PMCID: PMC3075066.
2. Balio CP, Wiley KK Jr, Greene MS, Vest JR. Opioid-Related Emergency Department Encounters: Patient, Encounter, and Community Characteristics Associated With Repeated Encounters. *Ann Emerg Med*. 2020 May;75(5):568-575. doi: 10.1016/j.annemergmed.2019.12.005. Epub 2020 Jan 23. PMID: 31983498.
3. Vivolo-Kantor AM, Seth P, Gladden RM, Mattson CL, Baldwin GT, Kite-Powell A, Coletta MA. Vital Signs: Trends in Emergency Department Visits for Suspected Opioid Overdoses - United States, July 2016-September 2017. *MMWR Morb Mortal Wkly Rep*. 2018 Mar 9;67(9):279-285. doi: 10.15585/mmwr.mm6709e1. PMID: 29518069; PMCID: PMC5844282.
4. Houry D, Adams J. Emergency Physicians and Opioid Overdoses: A Call to Aid. *Ann Emerg Med*. 2019 Sep;74(3):436-438. doi: 10.1016/j.annemergmed.2019.07.020. Epub 2019 Aug 8. PMID: 31402152; PMCID: PMC6945117.

HAN Archive - 00438 | Health Alert Network (HAN). (2021). Retrieved 12 February 2021, from https://emergency.cdc.gov/han/2020/han00438.asp

1. Johnson E, Lam C, Axeen S, Vosooghi A, Schneberk T. 28EMF The Opioid Epidemic Meets the Coronavirus Pandemic: Rates and Patient Characteristics of Emergency Department Visits for Opiate Use Disorder During the COVID-19 Pandemic in the Los Angeles County Public Hospital System. *Ann Emerg Med*. 2021;78(2):S14. doi:10.1016/j.annemergmed.2021.07.029
2. Weiner SG, Baker O, Bernson D, Schuur JD. One-Year Mortality of Patients After Emergency Department Treatment for Nonfatal Opioid Overdose. *Ann Emerg Med*. 2020 Jan;75(1):13-17. doi: 10.1016/j.annemergmed.2019.04.020. Epub 2019 Jun 20. PMID: 31229387; PMCID: PMC6920606.

Ashburn NP, Ryder CW, Angi RM, Snavely AC, Nelson RD, Bozeman WP, McGinnis HD, Winslow JT, Stopyra JP. One-Year Mortality and Associated Factors in Patients Receiving Out-of-Hospital Naloxone for Presumed Opioid Overdose. *Ann Emerg Med*. 2020 May;75(5):559-567. doi: 10.1016/j.annemergmed.2019.11.022. Epub 2020 Jan 23. PMID: 31983499.

1. D'Onofrio G, O'Connor PG, Pantalon MV, Chawarski MC, Busch SH, Owens PH, Bernstein SL, Fiellin DA. Emergency department-initiated buprenorphine/naloxone treatment for opioid dependence: a randomized clinical trial. *JAMA*. 2015 Apr 28;313(16):1636-44. doi: 10.1001/jama.2015.3474. PMID: 25919527; PMCID: PMC4527523.
2. Dunkley CA, Carpenter JE, Murray BP, Sizemore E, Wheatley M, Morgan BW, Moran TP, Steck A. Retrospective Review of a Novel Approach to Buprenorphine Induction in the Emergency Department. *J Emerg Med*. 2019 Aug;57(2):181-186. doi: 10.1016/j.jemermed.2019.03.029. Epub 2019 May 3. PMID: 31060846.
3. Kaucher KA, Caruso EH, Sungar G, Gawenus L, Hurlbut K, Sanchez DC, Broderick K. Evaluation of an emergency department buprenorphine induction and medication-assisted treatment referral program. *Am J Emerg Med*. 2020 Feb;38(2):300-304. doi: 10.1016/j.ajem.2019.158373. Epub 2019 Jul 30. PMID: 31387811.
4. Kelly T, Hoppe JA, Zuckerman M, Khoshnoud A, Sholl B, Heard K. A novel social work approach to emergency department buprenorphine induction and warm hand-off to community providers. *Am J Emerg Med*. 2020 Jun;38(6):1286-1290. doi: 10.1016/j.ajem.2019.12.038. Epub 2020 Jan 7. PMID: 31959523.
5. Busch SH, Fiellin DA, Chawarski MC, Owens PH, Pantalon MV, Hawk K, Bernstein SL, O'Connor PG, D'Onofrio G. Cost-effectiveness of emergency department-initiated treatment for opioid dependence. *Addiction*. 2017 Nov;112(11):2002-2010. doi: 10.1111/add.13900. Epub 2017 Aug 16. PMID: 28815789; PMCID: PMC5657503.
6. Wax PM, Stolbach AI, Schwarz ES, Warrick BJ, Wiegand TJ, Nelson LS. ACMT Position Statement: Buprenorphine Administration in the Emergency Department. *J Med Toxicol*. 2019 Jul;15(3):215-216. doi: 10.1007/s13181-019-00712-3. Epub 2019 May 13. PMID: 31087272; PMCID: PMC6597747.
7. American College of Emergency Physicians. (2017). Opioid Patients in the Emergency Department [PDF]. https://www.emergencyphysicians.org/globalassets/files/pdfs/opioid-poll-results-acep17.pdf

Wilson N, Kariisa M, Seth P, et al. [Drug and Opioid-Involved Overdose Deaths—United States, 2017-2018](https://www.cdc.gov/mmwr/volumes/69/wr/mm6911a4.htm). *MMWR Morb Mortal Wkly Rep* 2020;69:290-297

1. Hawk KF, D'Onofrio G, Chawarski MC, et al. Barriers and Facilitators to Clinician Readiness to Provide Emergency Department-Initiated Buprenorphine. *JAMA Netw Open*. 2020;3(5):e204561. Published 2020 May 1. doi:10.1001/jamanetworkopen.2020.4561
2. Lowenstein M, Kilaru A, Perrone J, Hemmons J, Abdel-Rahman D, Meisel ZF, Delgado MK. Barriers and facilitators for emergency department initiation of buprenorphine: A physician survey. *Am J Emerg Med*. 2019 Sep;37(9):1787-1790. doi: 10.1016/j.ajem.2019.02.025. Epub 2019 Feb 18. PMID: 30803850; PMCID: PMC7556325.
3. Grimshaw JM, Eccles MP, Walker AE, Thomas RE. Changing physicians' behavior: what works and thoughts on getting more things to work. *J Contin Educ Health Prof*. 2002 Fall;22(4):237-43. doi: 10.1002/chp.1340220408. PMID: 12613059.
4. Glanz K, Bishop DB. The role of behavioral science theory in development and implementation of public health interventions. *Annu Rev Public Health*. 2010;31:399-418. doi: 10.1146/annurev.publhealth.012809.103604. PMID: 20070207.
5. Ceccato NE, Ferris LE, Manuel D, Grimshaw JM. Adopting health behavior change theory throughout the clinical practice guideline process*. J Contin Educ Health Prof*. 2007 Fall;27(4):201-7. doi: 10.1002/chp.138. PMID: 18085640.
6. Armitage CJ, Conner M. Efficacy of the Theory of Planned Behaviour: a meta-analytic review. *Br J Soc Psychol*. 2001 Dec;40(Pt 4):471-99. doi: 10.1348/014466601164939. PMID: 11795063.
7. Ajzen, I. "The theory of planned behavior." Organizational behavior and human decision processes 50.2 (1991): 179-211.
8. Millstein SG. Utility of the theories of reasoned action and planned behavior for predicting physician behavior: a prospective analysis. *Health Psychol*. 1996;15(5):398‐402. doi:10.1037//0278-6133.15.5.398
9. Walker AE, Grimshaw JM, Armstrong EM. Salient beliefs and intentions to prescribe antibiotics for patients with a sore throat. *Br J Health Psychol*. 2001 Nov;6(Part 4):347-360. doi: 10.1348/135910701169250. PMID: 12614509.
10. Limbert, C, Lamb, R. Doctors' use of clinical guidelines: Two applications of the Theory of Planned Behaviour. *Psychology, health & medicine*. 2010;7(3):301-310. doi:10.1080/13548500220139377
11. Perez R, Brehaut JC, Taljaard M, Stiell IG, Clement CM, Grimshaw J. Theory of planned behaviour can help understand processes underlying the use of two emergency medicine diagnostic imaging rules. *Implement Sci*. 2014;9:88. Published 2014 Aug 7. doi:10.1186/s13012-014-0088-x
12. Madsen TE, Riese A, Choo EK, Ranney ML. Effects of a web-based educational module on pediatric emergency medicine physicians' knowledge, attitudes, and behaviors regarding youth violence. *West J Emerg Med*. 2014 Aug;15(5):615-22. doi: 10.5811/westjem.2014.4.21365. Epub 2014 Aug 1. PMID: 25157311; PMCID: PMC4140206.
13. Samuels EA, Dwyer K, Mello MJ, Baird J, Kellogg AR, Bernstein E. Emergency Department-based Opioid Harm Reduction: Moving Physicians From Willing to Doing. *Acad Emerg Med*. 2016 Apr;23(4):455-65. doi: 10.1111/acem.12910. Epub 2016 Mar 22. PMID: 26816030.
14. Choo EK, DeMayo RF, Sun BC. Is there a mismatch between policies to curtail physician opioid prescribing and what we know about changing physician behavior? *Int J Drug Policy*. 2018 Jun;56:54-55. doi: 10.1016/j.drugpo.2018.03.002. Epub 2018 Mar 19. PMID: 29567466.

Im DD, Chary A, Condella AL, et al. Emergency Department Clinicians' Attitudes Toward Opioid Use Disorder and Emergency Department-initiated Buprenorphine Treatment: A Mixed-Methods Study. *West J Emerg Med*. 2020;21(2):261-271. Published 2020 Feb 21. doi:10.5811/westjem.2019.11.44382

Crist RC, Reiner BC, Berrettini WH. A review of opioid addiction genetics. Curr Opin *Psychol*. 2019 Jun;27:31-35. doi: 10.1016/j.copsyc.2018.07.014. Epub 2018 Aug 9. PMID: 30118972; PMCID: PMC6368898.

Levis SC, Mahler SV, Baram TZ. The Developmental Origins of Opioid Use Disorder and Its Comorbidities. *Front Hum Neurosci*. 2021 Feb 11;15:601905. doi: 10.3389/fnhum.2021.601905. PMID: 33643011; PMCID: PMC7904686.

Chandler RK, Fletcher BW, Volkow ND. Treating drug abuse and addiction in the criminal justice system: improving public health and safety [published correction appears in JAMA. 2009 Mar 11;301(10):1024]. *JAMA*. 2009;301(2):183-190. doi:10.1001/jama.2008.976

Kopak AM. Breaking the addictive cycle of the system: improving US criminal justice practices to address substance use disorders. *Int J Prison Health*. 2015;11(1):4-16. doi: 10.1108/IJPH-07-2014-0023. PMID: 25751703.

Kelly JF, Dow SJ, Westerhoff C. Does Our Choice of Substance-Related Terms Influence Perceptions of Treatment Need? An Empirical Investigation with Two Commonly Used Terms. *Journal of Drug Issues*. 2010;40(4):805-818. doi:[10.1177/002204261004000403](https://doi.org/10.1177/002204261004000403)

Strayer, Reuben. “Simulation Package for ED Management of Opioid Use Disorder.” Emupdates, 24 Sept. 2020, emupdates.com/oudsim/.

Words Matter - Terms to Use and Avoid When Talking About Addiction. National Institute on Drug Abuse, 23 Oct. 2020, [www.drugabuse.gov/nidamed-medical-health-professionals/health-professions-education/words-matter-terms-to-use-avoid-when-talking-about-addiction](http://www.drugabuse.gov/nidamed-medical-health-professionals/health-professions-education/words-matter-terms-to-use-avoid-when-talking-about-addiction).

Zuckerman M, Kelly T, Heard K, Zosel A, Marlin M, Hoppe JA. Physician attitudes on buprenorphine induction in the emergency department: results from a multistate survey. *Clinical toxicology (Philadelphia, Pa)*. Published online 2020:1-7. doi:10.1080/15563650.2020.1805461

HHS Releases New Buprenorphine Practice Guidelines, Expanding Access to Treatment for Opioid Use Disorder. (2021). Retrieved 4 May 2021, from https://www.hhs.gov/about/news/2021/04/27/hhs-releases-new-buprenorphine-practice-guidelines-expanding-access-to-treatment-for-opioid-use-disorder.html

Fiscella K, Wakeman SE, Beletsky L. Buprenorphine Deregulation and Mainstreaming Treatment for Opioid Use Disorder: X the X Waiver. *JAMA Psychiatry*. 2019 Mar 1;76(3):229-230. doi: 10.1001/jamapsychiatry.2018.3685. PMID: 30586140.

1. Frank JW, Wakeman SE, Gordon AJ. No end to the crisis without an end to the waiver. *Subst Abus*. 2018;39(3):263-265. doi: 10.1080/08897077.2018.1543382. PMID: 30676296.
2. Heimer R, Hawk K, Vermund SH. Prevalent Misconceptions About Opioid Use Disorders in the United States Produce Failed Policy and Public Health Responses. *Clin Infect Dis*. 2019;69(3):546-551. doi:10.1093/cid/ciy977

*Clinical Opiate Withdrawal Scale*, National Institute on Drug Abuse, 2020, www.drugabuse.gov/sites/default/files/ClinicalOpiateWithdrawalScale.pdf.

Herring AA, Vosooghi AA, Luftig J, et al. High-Dose Buprenorphine Induction in the Emergency Department for Treatment of Opioid Use Disorder. *JAMA Netw Open.* 2021;4(7):e2117128. doi:10.1001/jamanetworkopen.2021.17128

Strain EC, Preston KL, Liebson IA, Bigelow GE. Buprenorphine effects in methadone-maintained volunteers: effects at two hours after methadone. *J Pharmacol Exp Ther*. 1995 Feb;272(2):628-38. PMID: 7853176.

Herring AA, Schultz CW, Yang E, Greenwald MK. Rapid induction onto sublingual buprenorphine after opioid overdose and successful linkage to treatment for opioid use disorder. *Am J Emerg Med*. 2019 Dec;37(12):2259-2262. doi: 10.1016/j.ajem.2019.05.053. Epub 2019 May 29. PMID: 31239086.

Martin A, Baugh J, Chavez T, Leifer J, Kao LS, Dutta S, White B, Hayes BD, Williamson D, Raja A. Clinician experience of nudges to increase ED OUD treatment. *Am J Emerg Med*. 2020 Mar 26:S0735-6757(20)30192-3. doi: 10.1016/j.ajem.2020.03.050. Epub ahead of print. PMID: 32546321.

Foster SD, Lee K, Edwards C, Pelullo AP, Khatri UG, Lowenstein M, Perrone J. Providing Incentive for Emergency Physician X-Waiver Training: An Evaluation of Program Success and Postintervention Buprenorphine Prescribing. *Ann Emerg Med*. 2020 Aug;76(2):206-214. doi: 10.1016/j.annemergmed.2020.02.020. Epub 2020 May 4. PMID: 32376089.

Rubin R. As Overdoses Climb, Emergency Departments Begin Treating Opioid Use Disorder. *JAMA*. 2018 Jun 5;319(21):2158-2160. doi: 10.1001/jama.2018.4648. PMID: 29800009.

Strayer RJ, Hawk K, Hayes BD, et al. Management of Opioid Use Disorder in the Emergency Department: A White Paper Prepared for the American Academy of Emergency Medicine. *The Journal of emergency medicine*. 2020;58(3):522-546. doi:10.1016/j.jemermed.2019.12.034

Herring AA, Perrone J, Nelson LS. Managing Opioid Withdrawal in the Emergency Department With Buprenorphine. *Ann Emerg Med*. 2019 May;73(5):481-487. doi: 10.1016/j.annemergmed.2018.11.032. Epub 2019 Jan 5. PMID: 30616926.

Ahmed OM, Mao JA, Holt SR, Hawk K, D'Onofrio G, Martel S, Melnick ER. A scalable, automated warm handoff from the emergency department to community sites offering continued medication for opioid use disorder: Lessons learned from the EMBED trial stakeholders. *J Subst Abuse Treat*. 2019 Jul;102:47-52. doi: 10.1016/j.jsat.2019.05.006. Epub 2019 May 7. PMID: 31202288; PMCID: PMC6578846.

Holland, Wesley C, Nath, Bidisha, Li, Fangyong, et al. Interrupted Time Series of User‐centered Clinical Decision Support Implementation for Emergency Department–initiated Buprenorphine for Opioid Use Disorder. *Academic emergency medicine*. 2020;27(8):753-763. doi:10.1111/acem.14002

Melnick, Edward R, Nath, Bidisha, Ahmed, Osama M, et al. Progress Report on EMBED: A Pragmatic Trial of User-Centered Clinical Decision Support to Implement EMergency Department-Initiated Buprenorphine for Opioid Use Disorder. *Journal of psychiatry and brain science*. 2020;5. doi:10.20900/jpbs.20200003

Andrilla CHA, Moore TE, Patterson DG, Larson EH. Geographic Distribution of Providers With a DEA Waiver to Prescribe Buprenorphine for the Treatment of Opioid Use Disorder: A 5-Year Update. *J Rural Health*. 2019 Jan;35(1):108-112. doi: 10.1111/jrh.12307. Epub 2018 Jun 20. PMID: 29923637.

Joudrey PJ, Edelman EJ, Wang EA. Drive Times to Opioid Treatment Programs in Urban and Rural Counties in 5 US States. *JAMA*. 2019 Oct 1;322(13):1310-1312. doi: 10.1001/jama.2019.12562. PMID: 31573628; PMCID: PMC6777265.

Grimm C. Geographic Disparities Affect Access to Buprenorphine Services for Opioid Use Disorder. Washington, D. C.: U.S. Department of Health and Human Services. Office of Inspector General; January 2020.

Goedel WC, Shapiro A, Cerda M, Tsai JW, Hadland SE, Marshall BDL. Association of Racial/Ethnic Segregation With Treatment Capacity for Opioid Use Disorder in Counties in the United States. *JAMA Netw Open.* 2020;3(4):e203711.

Snow RL, Simon RE, Jack HE, Oller D, Kehoe L, Wakeman SE. Patient experiences with a transitional, low-threshold clinic for the treatment of substance use disorder: A qualitative study of a bridge clinic. *Journal of substance abuse treatment.* 2019;107:1-7.

Langabeer JR 2nd, Yatsco A, Champagne-Langabeer T. Telehealth sustains patient engagement in OUD treatment during COVID-19. *J Subst Abuse Treat*. 2021 Mar;122:108215. doi: 10.1016/j.jsat.2020.108215. Epub 2020 Nov 24. PMID: 33248863; PMCID: PMC7685137.

Zheng W, Nickasch M, Lander L, et al. Treatment Outcome Comparison Between Telepsychiatry and Face-to-face Buprenorphine Medication-assisted Treatment for Opioid Use Disorder: A 2-Year Retrospective Data Analysis. *J Addict Med.* 2017;11(2):138-144.

Yang YT, Weintraub E, Haffajee RL. Telemedicine's Role in Addressing the Opioid Epidemic. *Mayo Clin Proc.* 2018;93(9):1177-1180.

Guille C, Simpson AN, Douglas E, et al. Treatment of Opioid Use Disorder in Pregnant Women via Telemedicine: A Nonrandomized Controlled Trial. *JAMA Netw Open.* 2020;3(1):e1920177.

Eibl JK, Gauthier G, Pellegrini D, et al. The effectiveness of telemedicine-delivered opioid agonist therapy in a supervised clinical setting. *Drug Alcohol Depend.* 2017;176:133-138.

National Institute on Drug Abuse. Medications to Treat Opioid Use Disorder Research Report: How much does opioid treatment cost?<https://www.drugabuse.gov/publications/research-reports/medications-to-treat-opioid-addiction/how-much-does-opioid-treatment-cost>. Published June 2018. Accessed September 24, 2020.

Beetham T. Buprenorphine Prior Authorization Removal: Low Hanging Fruit in the Opioid Overdose Crisis. *Harvard Public Health Review.* 2019;25.

Mark TL, Parish WJ, Zarkin GA. Association of Formulary Prior Authorization Policies With Buprenorphine-Naloxone Prescriptions and Hospital and Emergency Department Use Among Medicare Beneficiaries. *JAMA Netw Open.* 2020;3(4):e203132.

Muzyk A, Smothers ZPW, Collins K, MacEachern M, Wu LT. Pharmacists' attitudes toward dispensing naloxone and medications for opioid use disorder: A scoping review of the literature. *Subst Abus.* 2019;40(4):476-483.

Bach P, Hartung D. Leveraging the role of community pharmacists in the prevention, surveillance, and treatment of opioid use disorders. *Addict Sci Clin Pract.* 2019;14(1):30.

Feldman N. It's The Go-To Drug To Treat Opioid Addiction. Why Won't More Pharmacies Stock It? National Publiv Radio.<https://www.npr.org/sections/health-shots/2019/08/13/741113454/its-the-go-to-drug-for-opioid-addiction-so-why-won-t-more-pharmacists-stock-it>. Published August 13, 2019. Accessed September 25, 2020.

Centers for Medicare and Medicaid Services. Screening, Brief Intervention, and Referral to Treatment (SBIRT)  Services.<https://www.cms.gov/Outreach-and-Education/Medicare-Learning-Network-MLN/MLNProducts/downloads/SBIRT_Factsheet_ICN904084.pdf>. Published March 2020. Accessed September 22, 2020.

Kaiser Family Foundation. Medicaid Behavioral Health Services: Peer Support Services.<https://www.kff.org/other/state-indicator/medicaid-behavioral-health-services-peer-support-services/?currentTimeframe=0&sortModel=%7B%22colId%22:%22Location%22,%22sort%22:%22asc%22%7D>. Published 2018. Accessed September 21, 2020.

Centers for Medicare and Medicaid Services. Medicare Program; CY 2020 Revisions to Payment Policies Under the Physician Fee Schedule and Other Changes to Medicare Enrollment of Opioid Treatment Programs and Enhancements to Provider Enrollment Regulations Concerning Improper Prescribing and Patient Harm.<https://www.govinfo.gov/content/pkg/FR-2019-08-14/pdf/2019-16041.pdf>. Published August 14, 2019. Accessed September 23, 2020.

Friedman VE. Re: Request for Information for the Development of a CMS Action Plan to Prevent Opioid Addiction and Enhance Access to Medication-Assisted Treatment. American College of Emergency Physicians.<https://www.acep.org/globalassets/sites/acep/media/advocacy/federal-advocacy-pdfs/acep-response-to-cms-action-plan-to-prevent-opioid-addiction-and-enhance-access-to-mat.pdf>. Published October 11, 2019. Accessed September 23, 2020.

Colorado ACEP. *Opioid Prescribing & Treatment Guidelines.* Northglenn, CO: Colorado ACEP; 2017.

ED-BRIDGE. ED-BRIDGE Buprenorphine Guide. [https://ed-bridge.org](https://ed-bridge.org/). Published August 2018. Accessed June 1, 2019.

1. Kilaru AS, Perrone J, Kelley D, et al. Participation in a Hospital Incentive Program for Follow-up Treatment for Opioid Use Disorder. *JAMA Netw Open.* 2020;3(1):e1918511.

Pennsylvania Department of Human Services. Follow-up treatment after ED visit for Opioid Use Disorder. Hospital Quality Improvement Program Web site.<https://www.pamedsoc.org/docs/librariesprovider2/pamed-documents/new-opioid-measures-hqip.pdf?sfvrsn=8524485b_2>. Published June 6, 2018. Accessed June 1, 2019.

Baltimore City Health Department. Levels of Care for Baltimore City Hospitals Responding to the Opioid Epidemic Guide for Hospitals.<https://health.baltimorecity.gov/sites/default/files/Levels%20of%20Care%20-%20Guide.pdf>. Published August 2018. Accessed September 20, 2020.

NYC Department of Health and Mental Hygiene. Guidance for the Care of Patients Presenting to New York City Emergency Departments Following a Non-Fatal Opioid Overdose. NYC Department of Health and Mental Hygiene.<https://www1.nyc.gov/assets/doh/downloads/pdf/basas/non-fatal-overdose-providers.pdf>. Published May 31, 2019. Accessed June, 2019.

Samuels EA, McDonald JV, McCormick M, Koziol J, Friedman C, Alexander-Scott N. Emergency Department and Hospital Care for Opioid Use Disorder: Implementation of Statewide Standards in Rhode Island, 2017-2018. *Am J Public Health.* 2019;109(2):263-266.

Massachusetts Health and Hospital Association. Guidelines for Medication for Addiction Treatment for Opioid Use Disorder within the Emergency Department. Massachusetts Health and Hospital Association.<https://www.mhalink.org/MHADocs/MondayReport/2019/18-01-04MATguidelinesNEWFINAL.pdf>. Published January 2019. Accessed June 1, 2019.

1. American College of Emergency Physicians. ED X-Waiver Training Corps.<https://www.acep.org/education/ed-x-waiver-training-corps/>. Accessed September 25, 2020.
2. Ketcham E, Ryan R. BUPE: Buprenorphine use in the Emergency Department Tool. American College of Emergency Physicians.<https://www.acep.org/patient-care/bupe/>. Published 2018. Accessed Aug 1, 2019.
3. American College of Emergency Physicians. E-QUAL Network Opioid Initiative. https://www.acep.org/administration/quality/equal/emergency-quality-network-e-qual/e-qual-opioid-initiative/. Published 2019. Accessed June, 2019.
